# Special Issue: “Microstructures and Durability of Cement-Based Materials”

**DOI:** 10.3390/ma14040866

**Published:** 2021-02-11

**Authors:** Jeong Gook Jang, Solmoi Park

**Affiliations:** 1 Division of Architecture and Urban Design, Institute of Urban Science, Incheon National University, 119 Academy-ro, Yeonsu-gu, Incheon 22012, Korea; 2 Department of Civil Engineering, Pukyong National University, 45 Yongso-ro, Nam-gu, Busan 48513, Korea

**Keywords:** cement-based materials, alternative binders, microstructure, durability, characterization of materials

## Abstract

Cement-based materials play an irreplaceable role in building and sustaining our society by meeting the performance demand imposed on structures and sustainability. Cement-based materials are no longer limited to derivatives of Portland cement, and appreciate a wider range of binders that come from various origins. It is therefore of utmost importance for understanding and expanding the relevant knowledge on their microstructure and likely durability performance. This Special Issue “Microstructures and Durability of Cement-Based Materials” presents recent studies reporting microstructural and durability investigation revealing the characteristics of cement-based materials.

Concrete is the most widely used construction material and is also the second most consumed substance on the planet after water [1]. Portland cement is a typically used binder material for making concrete. The hydration of Portland cement governs the overall properties of concrete, therefore investigating the microstructure and durability of the cement, which is a prerequisite to understanding the performance of concrete. Recently, environmental and technical demands have been placed upon construction materials, requiring the development of materials with sustainability and enhanced performance. This has called for the use of new binder materials, as well as a thorough understanding and testing of such newly developed materials.

It is important to test and verify the performance of concrete and mortar prior to employing them in the relevant fields. A study by Fattah et al. [2] adopted a rapid chloride permeability test and bulk diffusion and sorption tests to identify porosity, sorptivity, salt diffusion and the permeability of concrete incorporating supplementary cementitious materials (SCMs) and chemical corrosion inhibitors. The laboratory test results were correlated with the field test obtained in the Arabian Gulf to conclude on the final concrete mixture that possesses outstanding resistance against chloride ingress. These findings are important because using SCMs as a partial replacement of Portland cement is necessary to mitigate the CO_2_ emissions associated with using concrete and it improves the overall performance by doing so. In a study by Li et al. [3], the authors investigated the intrinsic mechanism of fractures and damage induced by freeze–thaw in asphalt concrete and suggested the fracture model that can describe the fracture toughness during freeze–thaw cycles. The performance of cement-based materials can be evaluated by various tests as has been done by [2,3], while it can be investigated from a mechanistic viewpoint by employing various characterization techniques such as infrared spectroscopy, scanning electron microscopy [4], and backscattered electron image analysis [5]. By employing these characterization techniques, Zeng et al. [4] investigated the effect of temperature on the fluidity and early expansion characteristics of asphalt mortar, and Liu et al. [5] observed the hardening behavior of beta-C_2_S in a carbonation condition.

The performance of concrete can be improved by incorporating various admixtures, as demonstrated by [6], which investigated the durability and strength of casein-cemented sand incorporating slag. The use of fly ash is highlightable for enhancing the compression modulus of stabilized soil [7] and coastal soil [8]. In addition to typically employed and widely investigated SCMs, the use of other by-products can be beneficial. Seo et al. [9] demonstrated that the use of calcined oyster shell powder can be used as an expansive additive in cement-based materials, suggesting ~3 mass-% calcined oyster shell powder is recommendable. The use of crystalline waterproofing admixtures has been shown effective to inhibit unwanted crystal growth in hydrated cements [10].

SCMs can be used to partially replace Portland cement in concrete, or they can be solely used by alkali activation. A number of studies published in this Special Issue have investigated the performance of alkali-activated cements. Lee et al. [11] proposed an empirical model for predicting the age-dependent development of compressive and split tensile strength of alkali-activated fly ash and slag concretes. It is particularly useful to predict the effects of slag/binder, alkali solution/binder, silicate modulus and the NaOH concentration of the activator. Cho et al. [12] investigated the effect of cations on the sulfate resistance of alkali-activated fly ash and slag blended mortar, concluding that this binder tends to experience deleterious damage when magnesium sulfate solution is used. Kim et al. [13] reported that carbonation leads to a substantial decrease in the abrasion resistance of alkali-activated slag cements. Alkali-activated binders often exhibit a large extent of drying shrinkage, therefore Son et al. [14] explored a means of reducing the drying shrinkage by incorporating CaSO_4_ and investigating its effect on the pore structure. An unconventional means of producing alkali-activated cements has been presented in [15,16] where seawater has been used as mixing water for concrete, and calcium carbide residue has been used as an alkali activator.

The use of carbon nanomaterials in concrete is a topic for ongoing discussions. A study by Zheng et al. [17] showed that graphene oxide is effective for inducing calcite precipitation in hydrated C_3_S. In addition to the use of graphene in concrete, carbon nanotubes can be a useful admixture as demonstrated by Fan et al. [18], which showed that a carbon nanotube incorporated with styrene–acrylic emulsion additive in cement pastes leads to refining the pore structure and developing stronger interfacial adhesion and enhanced load transferability. The use of carbon nanotubes is also highlightable in terms of electromagnetic shielding performance, as they dramatically improve the electromagnetic shielding effectiveness of concrete when uniformly dispersed in a cement matrix [19].

## References

[1] Habert G., Miller S.A., John V.M., Provis J.L., Favier A., Horvath A., Scrivener K.L. (2020). Environmental Impacts and Decarbonation Strategies in the Cement and Concrete industries. Nat. Rev. Earth Environ..

[2] Abd El Fattah A., Al-Duais I., Riding K., Thomas M., Al-Dulaijan S., Al-Zahrani M. (2020). Field Validation of Concrete Transport Property Measurement Methods. Materials.

[3] Li J., Wang F., Yi F., Ma J., Lin Z. (2019). Fractal Analysis of the Fracture Evolution of Freeze-Thaw Damage to Asphalt Concrete. Materials.

[4] Zeng X., Zhu H., Lan X., Liu H., Umar H., Xie Y., Long G., Ma C. (2020). Effects of Temperature on Fluidity and Early Expansion Characteristics of Cement Asphalt Mortar. Materials.

[5] Liu S., Guan X., Zhang H., Wang Y., Gou M. (2019). Revealing the Microstructure Evolution and Carbonation Hardening Mechanism of β-C_2_S Pastes by Backscattered Electron Images. Materials.

[6] Park S.-S., Woo S.-W., Jeong S.-W., Lee D.-E. (2020). Durability and Strength Characteristics of Casein-Cemented Sand with Slag. Materials.

[7] Yang S., Liu W. (2019). The Effect of Changing Fly Ash Content on the Modulus of Compression of Stabilized Soil. Materials.

[8] Li N., Zhu Q., Wang W., Song F., An D., Yan H. (2019). Compression Characteristics and Microscopic Mechanism of Coastal Soil Modified with Cement and Fly Ash. Materials.

[9] Seo J.H., Park S.M., Yang B.J., Jang J.G. (2019). Calcined Oyster Shell Powder as an Expansive Additive in Cement Mortar. Materials.

[10] Azarsa P., Gupta R., Biparva A. (2020). Inventive Microstructural and Durability Investigation of Cementitious Composites Involving Crystalline Waterproofing Admixtures and Portland Limestone Cement. Materials.

[11] Lee S., Shin S. (2019). Prediction on Compressive and Split Tensile Strengths of GGBFS/FA Based GPC. Materials.

[12] Cho Y., Kim J.H., Jung S., Chung Y., Jeong Y. (2019). Importance of Cation Species during Sulfate Resistance Tests for Alkali-Activated FA/GGBFS Blended Mortars. Materials.

[13] Kim H.-K., Song K.-I., Song J.-K., Jang J.G. (2019). Effect of Carbonation on Abrasion Resistance of Alkali-Activated Slag with Various Activators. Materials.

[14] Son H., Park S.M., Seo J.H., Lee H.K. (2019). Effect of CaSO_4_ Incorporation on Pore Structure and Drying Shrinkage of Alkali-Activated Binders. Materials.

[15] Siddique S., Jang J.G. (2020). Mechanical Properties, Microstructure, and Chloride Content of Alkali-Activated Fly Ash Paste Made with Sea Water. Materials.

[16] Seo J., Park S., Yoon H.N., Jang J.G., Kim S.H., Lee H.K. (2019). Utilization of Calcium Carbide Residue Using Granulated Blast Furnace Slag. Materials.

[17] Zheng D., Yang H., Yu F., Zhang B., Cui H. (2019). Effect of Graphene Oxide on the Crystallization of Calcium Carbonate by C_3_S Carbonation. Materials.

[18] Fan J., Li G., Deng S., Deng C., Wang Z., Zhang Z. (2020). Effect of Carbon Nanotube and Styrene-Acrylic Emulsion Additives on Microstructure and Mechanical Characteristics of Cement Paste. Materials.

[19] Lee N., Kim S., Park G. (2019). The Effects of Multi-Walled Carbon Nanotubes and Steel Fibers on the AC Impedance and Electromagnetic Shielding Effectiveness of High-Performance, Fiber-Reinforced Cementitious Composites. Materials.

